# FACTORS ASSOCIATED WITH FRID USE IN OLDER BLACK AND WHITE MEN AND WOMEN: THE HEALTH ABC STUDY

**DOI:** 10.1093/geroni/igac059.3098

**Published:** 2022-12-20

**Authors:** Jimmie Roberts, Obert M Boudreau, Lingshu Xue, Jane A Cauley, Elsa Strotmeyer

**Affiliations:** School of Public Health, University of Pittsburgh, Pittsburgh, Pennsylvania, United States; School of Public Health, University of Pittsburgh, Pittsburgh, Pennsylvania, United States; School of Public Health, University of Pittsburgh, Pittsburgh, Pennsylvania, United States; School of Public Health, University of Pittsburgh, Pittsburgh, Pennsylvania, United States; School of Public Health, University of Pittsburgh, Pittsburgh, Pennsylvania, United States

## Abstract

Medications that increase falls, fall-risk increasing drugs (FRIDs), are common in older adults. Two FRID definitions, the CDC Steadi-Rx and Swedish National Board of Health and Welfare, are widely accepted. We hypothesized that FRID use risk factors vary by definition in 1,352 community-dwelling older adults in the Health, Aging, and Body Composition Study (Health ABC; 2007–2008 clinic visit; 83.4±2.8 years; 54.1% women; 34.9% Black). FRID use by either definition was associated with chronic health conditions, medical care including non-FRID use, higher BMI and depression scores, and less walking exercise (all p < 0.05). Steadi-Rx FRID use was also associated with more falls, ADL difficulty, and better cognitive scores. Using stepwise multivariate Poisson regression adjusting for demographics, lifestyle/behavior factors, and comorbidity, a 1-unit increase in BMI and depression score was associated with an approximately 2% mean FRID count increase for both definitions and 7% mean FRID count increase per 1-unit increase in non-FRID count. Both definitions had a 40% and 15% lower mean FRID count, respectively, with hypertension and cardiovascular disease (CVD) history. Better cognitive scores were associated with 1% mean increase in Steadi-Rx FRID count and a mean decrease of 13% and 33% FRID count, respectively, with cancer history and having primary healthcare. In identically adjusted logistic regression, FRID use (yes/no) was associated in a consistent direction with BMI, depression score, non-FRID count, hypertension, CVD, having primary healthcare, and also less likely with low-vs-high income (OR=0.18[0.06–0.50]). Risk factors differ by FRID definition, with Steadi-Rx identifying more predictors than the Swedish definition.

